# High-frequency percussive ventilation at altitude: study in a hypobaric chamber with a mechanical test lung

**DOI:** 10.1186/cc12061

**Published:** 2013-03-19

**Authors:** AC Cirodde, S Montmerle, ND Donat, CB Bourhillon, P Jault, L Bargues, T Leclerc

**Affiliations:** 1 Military Hospital Percy, Clamart, France; 2 IRBA, Brétigny, France

## Introduction

High-frequency percussive ventilation (HFPV) is a rescue technique for most severe acute lung injury/acute respiratory distress syndrome (ARDS) patients [1], especially with smoke inhalation or respiratory burns [2]. This study aimed at characterizing HFPV as delivered by Percussionnaire VDR4^® ^and at evaluating how hypobarism interferes with HFPV, in order to assess its usability at altitude.

## Methods

Using a mechanical test lung mimicking ARDS (compliance 17 ml/cmH_2_O) with two resistance levels (5 and 15 cmH_2_O/l/second) and ventilated with VDR4^® ^in a hypobaric chamber, ascents/descents between 0 and 5,000 and then 0 and 8,000 ft were performed. Adjustable VDR4^® ^parameters were modified one at a time at each altitude. Besides these parameters (cross-measured with standalone hardware), oxygen consumption of the respirator and three calculated parameters were studied: low-frequency tidal volume (Vt, integrated from instantaneous flows measured with a Fleisch pneumotachograph), end-inspiratory (PmEI) and end-expiratory (PmEE) mean pressures. PmEI and PmEE in HFPV reflect plateau pressure and positive end-expiratory pressure in conventional ventilation. The correction of altitude-induced offset with the modification of working pressure was also tested.

## Results

Data displayed by VDR4^® ^overestimated pulmonary pressures by more than 10%, but were reliable for other parameters. During ascent, an offset appeared for all respiratory parameters: Vt increased by 59% and PmEI by 53% between 0 and 8,000 ft. During descent, the offset was reversely directed with a 39% decrease in Vt and a 28% decrease in PmEE between 8,000 and 0 ft. Modifying working pressure adequately corrected PmEI and PmEE, but not Vt. In all cases, manually correcting VDR4^® ^parameters to their 0 ft level also corrected these offsets. Multivariate analysis further established that, adjusting for other parameters, Vt, PmEI and PmEE did practically not depend on altitude. Oxygen consumption of the respirator was high, 25 l/minute at 0 ft, and stable with altitude. It was reduced with percussive rate and with FiO_2_.

## Conclusion

HFPV can be safely used at altitude, provided that VDR4^® ^displayed parameters are used to manually adjust settings in order to avoid exposing patients to volutrauma or barotrauma during ascent, and to major hypoventilation and alveolar collapse during descent. The high oxygen consumption is currently the main limit to its use for long-range aeromedical evacuations.
